# Identification of key genes in pathogenesis of placental insufficiency intrauterine growth restriction

**DOI:** 10.1186/s12884-022-04399-3

**Published:** 2022-01-28

**Authors:** Chunhua Zhang, Jiao Ding, Hong Li, Ting Wang

**Affiliations:** 1 The Affiliated Suzhou Hospital of Nanjing Medical University, Suzhou, 215000 Jiangsu Province China; 2 The First Affiliated Hospital of Soochow University, Suzhou, 215006 Jiangsu Province China

**Keywords:** Intrauterine growth restriction, Placental insufficiency, Protein-protein interaction network, Weighted gene co-expression network analysis, Key gene

## Abstract

**Background:**

Intrauterine growth restriction (IUGR) is defined as a fetus that fails to achieve its genetically determined growth potential. The exact molecular mechanisms of placental insufficiency IUGR pathogenesis are a little known. Our goal was to identify key genes and gene co-expression modules related to placental insufficiency IUGR.

**Methods:**

We used weighted gene co-expression network analysis (WGCNA) and protein-protein interaction (PPI) network analysis to examine the IUGR dataset GSE114691 from NCBI Gene Expression Omnibus. Core modules and hub nodes of the protein-protein interaction network were identified. A gene network was constructed and genes were classified by WGCNA into different modules. The validation of potential key genes was carried out using additional datasets (GSE12216 and GSE24129).

**Results:**

We identified in GSE114691 539 down regulated genes and 751 up regulated genes in placental tissues characteristic of placental insufficiency IUGR compared with non-IUGR, and defined 76 genes as hub nodes in the protein-protein interaction network. Genes in the key modules of the WGCNA network were most closely associated with placental insufficiency IUGR and significantly enriched in biological process such as cellular metabolic process and macromolecule metabolic process. We identified as key genes TGFB1, LEP, ENG, ITGA5, STAT5A, LYN, GATA3, FPR1, TGFB2, CEBPB, KLF4, FLT1, and PNPLA2. The RNA expression levels of ENG and LEP, as biomarkers, were validated.

**Conclusion:**

A holistic gene expression profile of placental insufficiency IUGR has been generated and the key genes ENG and LEP has potential to serve as circulating diagnosis biomarkers and therapeutic targets for placental insufficiency IUGR.

**Supplementary Information:**

The online version contains supplementary material available at 10.1186/s12884-022-04399-3.

## Background

Intrauterine growth restriction is defined as a fetus that fails to achieve its genetically determined growth potential [1]. Intrauterine growth restriction is generally diagnosed as an estimated fetal weight less than the 10th percentile for gestational age [2, 3]. Intrauterine growth restriction is a major cause of neonatal neurological/respiratory morbidities and perinatal mortality [4, 5]. Intrauterine growth restriction can be related to maternal, fetal, placental and environmental factors. Maternal risk factors associated with IUGR include maternal diabetes, pre-existing hypertension, cyanotic heart disease, and obesity [6–8]. Intrauterine growth restriction is also associated with fetal genetic disorders such as chromosomal abnormalities, congenital abnormalities, and infection [9]. Placental or umbilical cord abnormalities, such as placenta previa, placental tumors, and cord anomalies, are important etiological factors that can contribute to IUGR; IUGR with such kind of clinical symptoms is usually classified as placental insufficiency IUGR [10–12]. To date, in normal obstetric practice, there has been little progress in detection, prevention, and treatment for placental insufficiency IUGR [13].

An estimated fetal weight less than the 10th percentile for gestational age, associated with an abnormal pulsatility index of the umbilical artery, is routinely used to diagnose IUGR and take measures to improve the outcome in the late stage of pregnancy [2, 14, 15]. These diagnostic criteria are still based on non-specific index and symptoms, with difficulty in timely and accurately identifying and managing placental insufficiency IUGR. At the serum protein level, the sFlt-1/PlGF ratio has been shown to be useful for predicting the real risk of developing IUGR among patients with sensitivity 44.4%, but the ratio needs to be used jointly with maternal factors (for example hypertension, diabetes) and ultrasound biometries to increase the sensitivity (84.1%) [16–18]. The occurrence of placental insufficiency IUGR has been reported to be correlated with activation of genes such as TGFβ, NOD1, LEP [19–21]. However, the development of placental insufficiency IUGR is a complex and progressive process, little is known about the exact molecular pathogenesis of placental insufficiency IUGR [22]; therefore, it remains of great significance to clarify genes contributing to placental insufficiency IUGR.

Many diseases develop systematically, and they are not driven by single genes. Placental insufficiency IUGR might be a complex disease caused by a group of special genes, thus, we hypothesized that it was important to investigate which key genes contribute to placental insufficiency IUGR by acting cooperatively as gene modules. High-throughput research methods and screening of important information form the basis for subsequent research. Weighted gene co-expression network analysis and protein-protein interaction network analysis are tools that focus on genome-wide expression [23]. WGCNA is used to identify potential gene modules related to disease traits based on the influence and regulation relationship between genes with similar expression patterns. PPI network analysis focuses on physical and functional associations based on data derived mainly from computational predictions, high-throughput experiments, automated text mining, and co-expression networks. Hence, both tools can be useful to identify candidate key genes or gene modules related to placental insufficiency IUGR [24].

In this study, we tried to identify key genes and its modules related to placental insufficiency IUGR by the section of genes obtained by PPI and WGCNA and validate them repeatedly, expecting to provide potential biomolecules for early detection at the circulating level, for subsequent clinical treatment, and to more fully understand the pathogenesis mechanism of placental insufficiency IUGR.

## Materials and methods microarray data

A statement to confirm that all methods were carried out in accordance with relevant guidelines and regulations (Declaration of Helsinki). The IUGR datasets GSE114691, GSE12216, and GSE24129 were obtained from NCBI Gene Expression Omnibus (GEO; https://www.ncbi.nlm.nih.gov/geo/). GSE114691 consists of 18 placental insufficiency IUGR samples and 21 normal samples, and the platform is GPL11154 Illumima HiSeq 2000 (*Homo sapiens*). GSE12216 contains eight placental insufficiency IUGR samples and eight normal samples, and its platform is GPL2986 ABI Human Genome Survey Microarray Version2. GSE24129 contains eight placental insufficiency IUGR samples and eight normal samples, and the platform is GPL6244 [HuGene-1_0st] Affymetrix Human Gene 1.0 ST Array [transcript (gene) version]. Characteristics of the studied populations in GSE114691, 12216, and 24129 met the following criteria (Supplementary Table 1): (1) intrauterine growth restriction with estimated fetal weight below the 10th percentile, (2) all IUGR individuals showed clinical signs of disturbed placental function such as asymmetric growth, oligohydroamnions, and/or increased pulsatility index of the umbilical artery, (3) known maternal and fetal factors were excluded, such as maternal systemic diseases (hypertension, diabetes, et.al, especially at the beginning of inclusion), multiple gestation, fetal congenital infection, structural abnormalities, and chromosomal abnormalities, (4) early and/or late onset IUGR [25–27].

### Differentially expressed gene screening

Differentially Expressed Genes (DEGs) between placental insufficiency IUGR and normal samples in the GSE114691 dataset were screened by the “edge” R package. For GSE12216 and GSE24129, DEGs were screened by the “limma” R package. The DEGs were considered statistically significant by the following criteria: (1) log2 (fold change) > 0.5 or log2 (fold change) < −0.5 (2) *P*-value < 0.05. The ggplot2 package in R was used to construct heat maps and volcano plots.

### Protein-protein interaction network construction and hub nodes selection

The PPI Network of DEGs in GSE114691 was constructed by Search Tool for the Retrieval of Interacting Genes (STRING ver. 11.0; http://string-db.org) online database. The PPI network of DEGs was statistically significant at the combined score > 0.4. The PPI networks were drawn using Cytoscape 3.7.2 [28], the core modules of the PPI were analyzed by MCODE (The Molecular Complex Detection), and the hub nodes were selected with degrees ≥30 calculated by cytoHubba.

### Weighted gene co-expression network analysis

The raw count data of GSE114691 were downloaded from the GEO database. A gene was defined as detectable if at least two samples contained more than one transcript from the gene. The CPM function was used to preprocess and normalize the raw data. All 23,206 genes were subjected to WGCNA (https://labs.genetics.ucla.edu/horvath/CoexpressionNetwork/Rpackages/WGCNA/Tutorials/). The R package WGCNA was used, and appropriate power parameter β was selected automatically by the pickSoft threshold function. The network was generated by turning adjacency into topological overlap by measuring the network connectivity of a gene defined as the sum of its adjacency with all other genes.

The hierarchical clustering dendrogram was constructed by classifying genes with similar expression profiles into modules based on the Topological Overlap Matrix dissimilarity with a minimum size of 20. The dissimilarity of module eigengenes was calculated to choose a cutline to merge some modules. The module eigengenes were obtained by the WGCNA to characterize the expression profiles of module genes. We calculated the correlation between eigengenes and clinical traits, and identified the relevant modules.

### Kyoto encyclopedia of genes and genomes and gene ontology enrichment analyses

The online database for Annotation, Visualization, and Integrated Discovery (DAVID, version 6.7; http://david.ncifcrf.gov) [29] was used to provide functional annotation information of genes for the Kyoto Encyclopedia of Genes and Genomes (KEGG) and Gene Ontology (GO) enrichment analyses. GO analyses showed the unique biological significance based on the screened genes. KEGG identified the important pathways involved in special phenotype.

### Identification and validation of key genes closely related to placental insufficiency IUGR

Venn diagrams (http://bioinformatics.psb.ugent.be/webtools/Venn/) were constructed between hub genes in PPI and epigenes in special module of WGCNA. Overlapped genes were considered candidate key genes closely correlated with placental insufficiency IUGR.

We used another GEO dataset, GSE12216, to validate the expression status of the key genes. We set the cutoff as log2 fold change>|0.5| and *P* < 0.05 to screen the DEGs. A volcano plot of DEGs and hierarchical clustering heat map of DEGs were constructed. A Venn diagram was constructed to overlap the upregulated/downregulated DEGs with the genes in positively /negatively related modules correspondingly. Transcription-level validation of key genes was performed with GSE24129.

## Results

### Protein-protein interaction network construction and module analysis

**Fig. 1:**
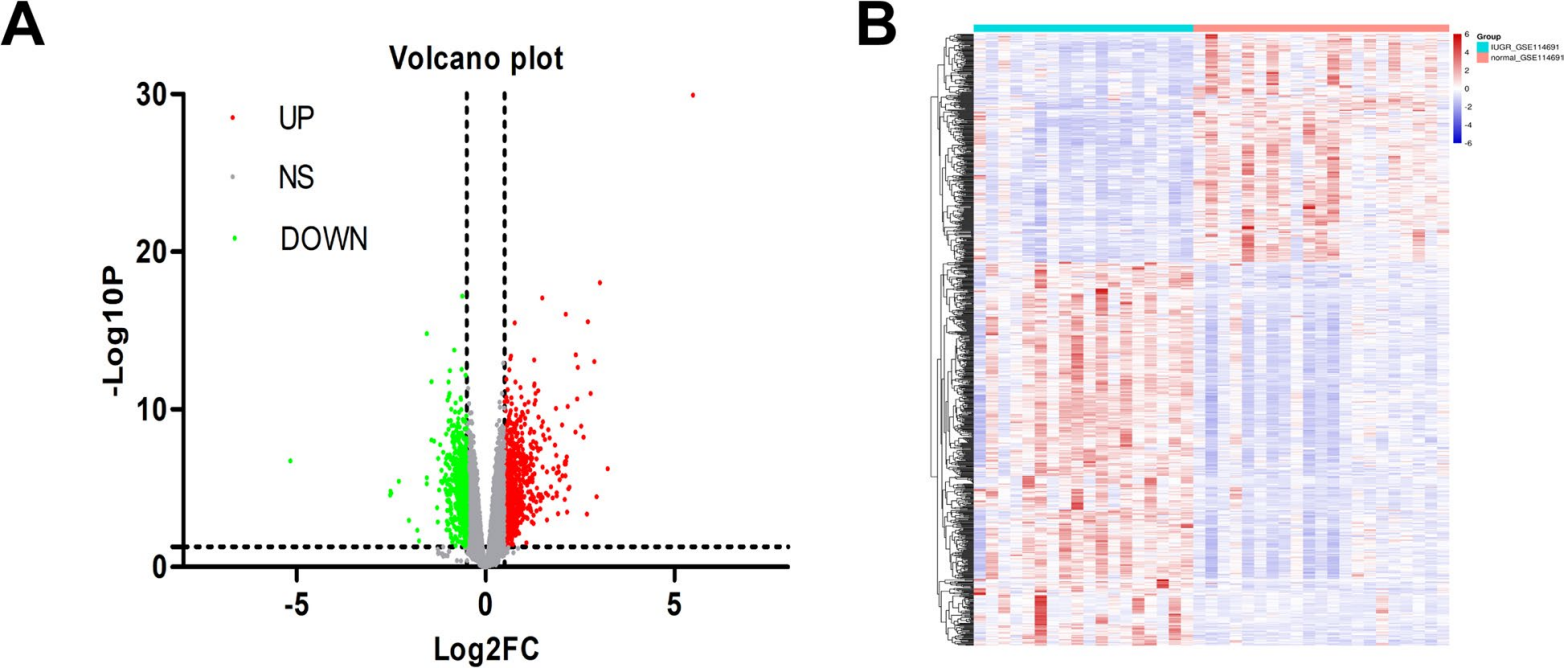
Volcano plot (**A**) and hierarchical clustering diagram (**B**) of differentially expressed genes in GSE114691

To identify the molecular basis for placental insufficiency IUGR pathogenesis, after standardization of the high-throughput sequencing data, we identified differentially expressed genes in GSE114691. We found 539 down regulated genes and 751 up regulated genes in placental tissues from pregnancy with placental insufficiency IUGR compared with non-IUGR tissues (Supplementary Table 2). Figure 1A, and B show the volcano plot and hierarchical clustering diagram heat map of these differentially expressed genes.

**Fig. 2:**
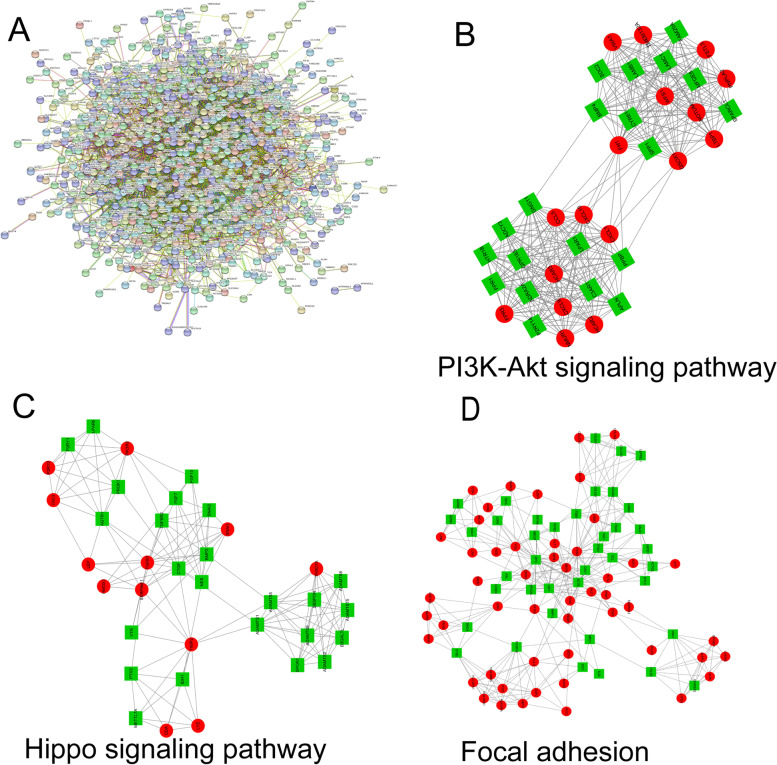
Protein-protein interaction networks obtained from STRING. (**A**) The PPI network constructed by STRING based on differentially expressed genes. (**B**, **C**, **D**) The 73 highest k-core genes make up three important subnetworks, PI3K-Akt signaling pathway, Hippo signaling pathway, and focal adhesion

To identify the functions of the DEGs, we constructed a protein-protein interaction network of the DEGs with 846 nodes and 4757 edges (Fig. 2A). To identify the key PPI network modules, we used the MCODE application from the Cytoscape software suite to perform gene network clustering analysis. The 73 highest k core genes comprised three important networks, the PI3K-Akt signaling pathway, the Hippo signaling pathway, and focal adhesion. These networks may suggest the basis for placental insufficiency IUGR pathogenesis (Fig. 2B, C, D).

Nodes with the greatest numbers of interactions with neighboring nodes were considered hub nodes. We used cytoHubba and identified 76 genes as hub nodes with degrees ≥30. Supplementary Table 3 shows the gene symbols, gene description, and degree for these hub nodes.

### Construction of the co-expression modules of placental insufficiency IUGR

**Fig. 3:**
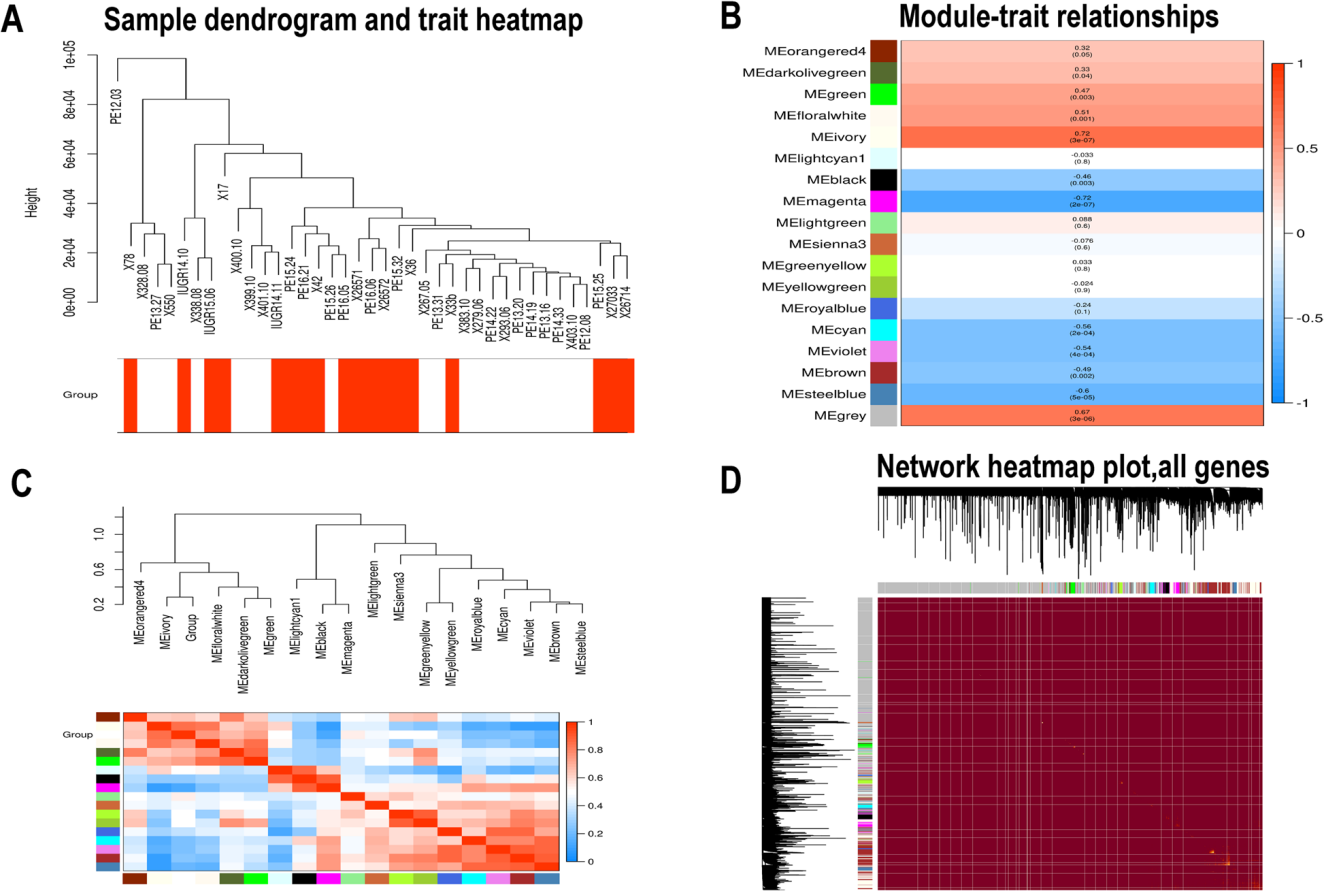
WGCNA results based on the expression data of GSE114691. (**A**) Sample dendrogram and trait heat map. The color corresponds to disease status (normal and placental insufficiency IUGR) (**B**) Heat map of the correlation between module eigengenes and the disease status of placental insufficiency IUGR. (**C**) Hierarchical clustering of module hub genes that summarize the modules yielded in the clustering analysis. Heat map plot of the adjacencies in the hub gene network. (**D**) Heat map plot shows the topological overlap matrix of all genes. Light color shows low overlap, and red color indicates high overlap. The left side and the top side show the gene dendrogram and module assignment

To classify key modules and genes in placental insufficiency IUGR, we used the WGCNA package to uncover highly correlated genes and co-expression networks. First, samples in GSE114691 were used for cluster analysis by the “hclust” package, for which the soft thresholding power value was chosen as 26 (β = 26, automatically by the pickSoftThreshold function). A hierarchical clustering tree (dendrogram, Fig. 3A) composed of 18 merged co-expression modules was produced.

### Gene co-expression modules correspond to clinical traits

Figure 3C shows interaction relationships of the 18 co-expression modules. Each co-expression module independently validated the others in the network. As shown in Fig. 3B, by Pearson’s correlation analysis, the module-feature relationship of the ivory module revealed a highly positive correlation (cor = 0.72, *P* = 2e-07) with placental insufficiency IUGR compared with other modules, whereas the magenta module illustrated that they were highly negatively correlated with placental insufficiency IUGR (cor = − 0.72, *p* = 2e-7). In addition, we constructed an eigengene dendrogram and heatmap to examine groups of correlated eigengenes and the dendrogram of all modules (Fig. 3D).

### Functional enrichment analysis in two key modules

**Fig. 4:**
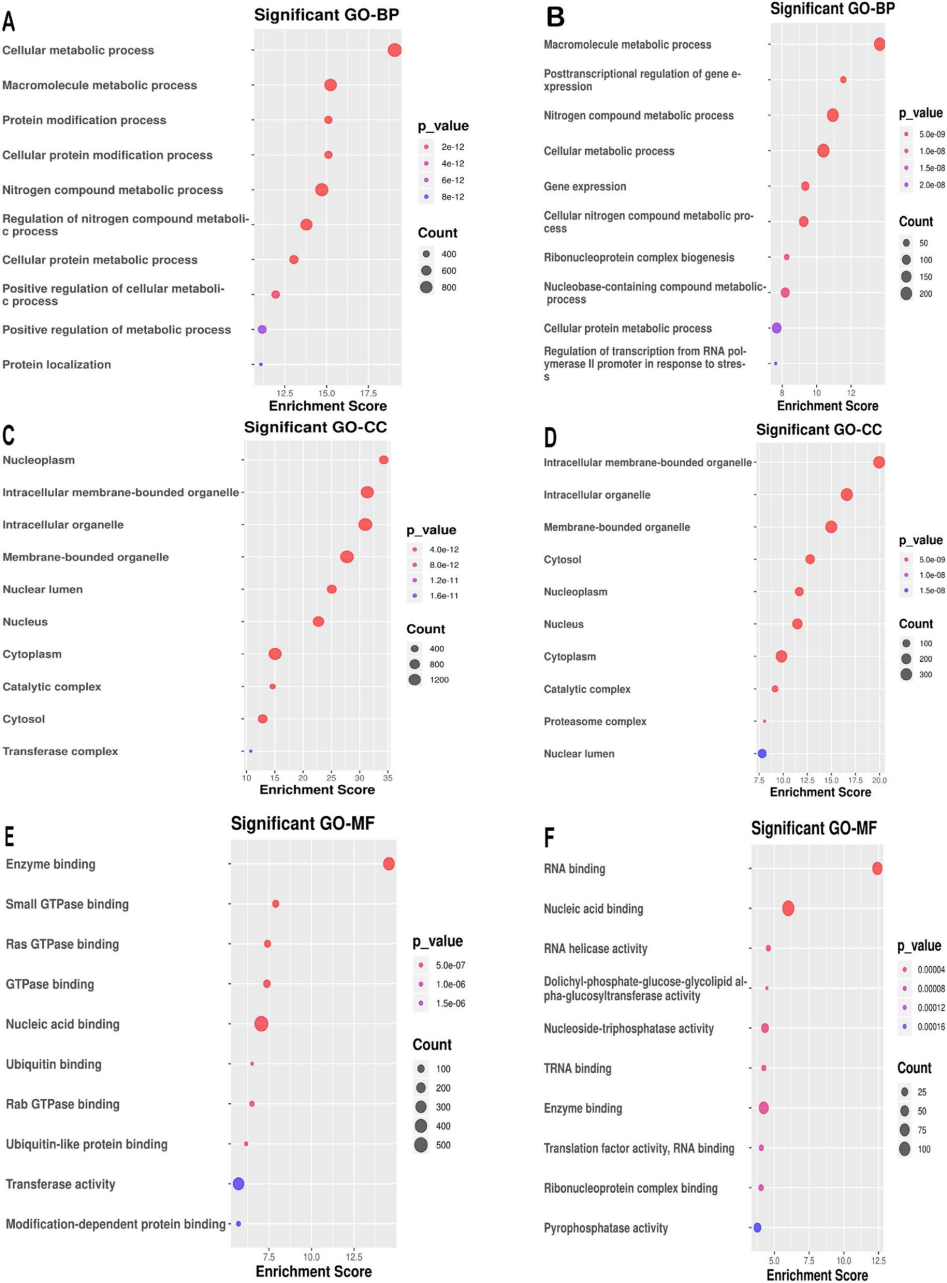
Gene Ontology enrichment analysis of genes in ivory and magenta modules obtained from WGCNA of GSE114691. (**A**, **C**, **E**) The top ten GO-BP (biological process), GO-CC (cellular component), GO-MF (molecular function) items of genes in the ivory module. (**B**, **D**, **F**) The top ten GO-BP, GO-CC, and GO-MF items of genes in magenta module

To understand the biological functions of gene sets involved in the ivory and magenta modules, we performed GO enrichment analysis (Fig. 4). The ivory module was significantly enriched in critical biological functions such as cellular metabolic process, nucleoplasm, and enzyme binding (Fig. 4A, C, E), which were correlated with pathogenesis of placental insufficiency IUGR. In the magenta module, the functions were concerned mainly with inhibition of placental insufficiency IUGR, significantly enriched in regulation of macromolecule metabolic process, intracellular membrane-bounded organelle, and RNA binding (Fig. 4B, D, F).

### Identification of key genes by overlapping analyses

**Fig. 5:**
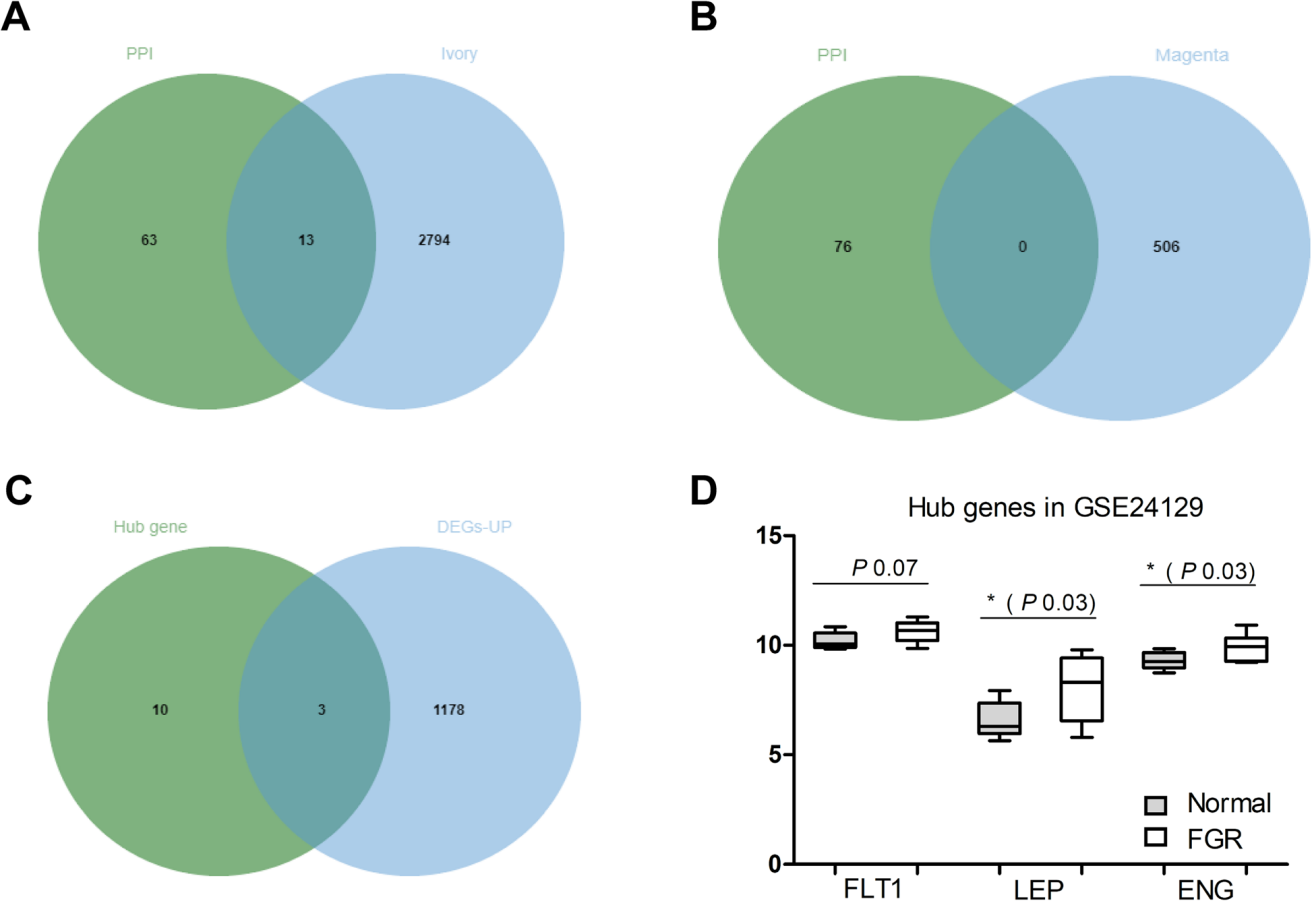
Identification and validation of key genes in GSE114691. (**A**) Overlap identification of common genes between hub nodes obtained in PPI and the ivory module. The 13 genes in the ivory module were also hub nodes in PPI. (**B**) Overlap identification of common genes between hub nodes obtained in PPI and the magenta module. (**C**) Overlap identification of common genes between upregulated DEGs in GSE12216 and the genes in ivory module of GSE114691. The three genes in the ivory module were also upregulated DEGs in GSE12216. (**D**) Validation of key genes at the transcriptional level in GSE24129

To identify the key genes related to placental insufficiency IUGR, we analyzed overlapping genes in GSE114691. Hub nodes identified in PPI network were used to construct a Venn diagram with genes in the ivory module of WGCNA. Thirteen mRNAs were present in the overlap area of the genes in the ivory module and hub nodes identified in the PPI network (Fig. 5A). No mRNAs were present in the overlap area of genes in the magenta module and hub nodes identified in PPI network (Fig. 5B). We identified the 13 overlapping DEGs as potential key genes for further analysis.

### Validation of the key genes

To validate the key genes identified by overlap analysis, we examined the 13 potential key genes in the GEO database GSE12216. Fig. S1A and S1B show the volcano plot and hierarchical clustering diagram heatmap of the differentially expressed genes in GSE12216. The expression of genes ENG, LEP, and FLT1 were significantly upregulated in placental insufficiency IUGR (Fig. 5C). This result indicated that the higher expression status of ENG, LEP, and FLT1 was consistent with placental insufficiency IUGR, namely, that the expression of the three key genes of the ivory module was positively correlated with the placental insufficiency IUGR. The relationship between the expression of these genes and the placental insufficiency IUGR status was further revealed by the transcriptional levels of ENG, LEP, and FLT1 obtained from the database GSE24129; ENG and LEP were expressed at significantly higher levels in placental insufficiency IUGR tissues compared with normal tissues (Fig. 5D). Thus, overexpression of ENG (*p* = 0.03) and LEP (*p* = 0.03) was significantly related to placental insufficiency IUGR status. The preliminary clinical value of mRNAs for ENG, LEP, and FLT1 was demonstrated with moderate diagnostic efficiency by the receiver operating characteristic curves as Fig. S2.

## Discussion

For IUGR caused by placental insufficiency, the clinical symptoms are observed by ultrasound in the late stages of pregnancy [27]. Instead of fetal size or growth trajectory, Doppler velocimetry evaluation of placental dysfunction might be a better diagnostic indicator of placental insufficiency IUGR [5, 30]. However, molecular biomarkers of placental insufficiency are expected to provide better detection rates even in the earlier pregnancy compared with diagnostic indices such as Doppler parameters. Genes in section of PPI network base on DEGs and module that positively/negatively related to pathogenesis of placental insufficiency IUGR were identified. ENG, LEP, and FLT1 were identified as key genes related to placental insufficiency IUGR, Significantly higher expression of ENG and LEP at RNA level in this disease was validated reliable by three GEO datasets repeatedly. (Fig. S3). Thus, ENG and LEP as biomarkers were detected and validated in the placental tissue from pregnancies complicated by placental insufficiency IUGR. These genes have potential to be clinical biomarkers at circulating level and will help future studies on treatment that target ENG and LEP.

There are few reports on ENG suggesting its mechanism in placental insufficiency IUGR. Endoglin as the product of ENG gene, a vascular endothelium glycoprotein, functions in the regulation of angiogenesis [31–33]. Thus, ENG might be related to placental insufficiency by interfering with the invasion of trophoblast-derived cells into maternal uterine tissue. Clinically higher amounts of circulating soluble endoglin are found in women with small for gestational age babies whose birth weight less than the 10% percentile [34].

The LEP gene encodes a peptide hormone (leptin) that regulates food intake and energy metabolism by inducing anorexinogenic factors and inhibiting orexigenic neuropeptides secretion [35]. The LEP protein also regulates bone mass and secretion of hypothalamo-pituitary-adrenal hormones [36, 37]. As for the human placenta, leptin is synthesized in the trophoblast and secreted to the maternal circulation, and secreted from placenta into fetal circulation [38]. The LEP protein might be involved in cell cycle regulation by activating signaling pathways such as JAK2STAT3, MAPK1/3, or PI3K-AKT1 (consistent with a predicted pathway in the part of PPI analysis), further functions in pathogenesis of placental insufficiency IUGR [39].

The FLT1 gene encodes a member of the vascular endothelial growth factor receptor (VEGFR) family, which functions in angiogenesis by binding to VEGFR-A, VEGFR-B, and placental growth factor [40]. Placental FLT1 overexpression suppresses the differentiation of the spongiotrophoblast into glycogen cells; thus, FLT1 are reported to functions in the intrauterine growth restriction phenotype as a result of the reduced exchange area of labyrinth, glycogen stores, and decreased expression of glucose transporter in an IUGR mouse model [41]. Based on that early/late onset IUGR and preeclampsia are sharing some common pathogenic genes, the ratio sFlt1/PIGF has been clinically applied to screen out IUGR (not only placental insufficiency IUGR) and preeclampsia at the maternal serum protein level [18].

Besides ENG, LEP, and FLT1 identified in this study, STAT3, long non-coding RNA H19, and HoxB7 has been reported to contribute to placental insufficiency IUGR by regulating the invasion and proliferation of trophoblasts by the necroptosis pathway RIPK1 and SIRT2, non-canonical TGF-β signaling pathway or Wnt1_β-catenin pathway [42–44]. Further analysis should be conducted to determine how other genes and pathways identified in this study contribute to placental insufficiency IUGR as well as what the clinical value of these genes used alone or jointly is.

### Limitations and strengths

This study relied on previously published datasets. The suggested pathogenic mechanisms of ENG, LEP, and FLT1 related to placental insufficiency IUGR needs further validation in cell and/or animal model. The datasets used in this study contain early and late onset cases of IUGR, with and without preeclampsia, which indicates the following endeavor to explore gene signatures for differentiating them. In addition, maternal ENG and LEP mRNA can be released to peripheral blood, and mix with RNA from placentas, different from placental specific gene FLT1, thus maternal parameters such as body mass index, age, and pregnant weeks should be considered when evaluating their clinical values of mRNAs (cutoff, sensitivity, specificity, Youden index) with large sample size at circulating level.

This study identified and validated ENG and LEP as key genes in the placental tissue from pregnancies complicated by placental insufficiency IUGR, with the clinical signs of asymmetric growth, oligohydroamnions, and/or increased pulsatility index of the umbilical artery. It suggests that mRNA of ENG and LEP have potential to be circulating biomarkers for diagnosis and therapeutic target in most instances of early and/or late onset placental insufficiency IUGR characteristic of the above clinical signs.

## Conclusion

Key genes identified by overlapping analysis of genes in key modules of WGCNA and hub nodes in PPI networks have important activities in pathogenesis of placental insufficiency IUGR. The key genes LEP and ENG have potential to serve as circulating diagnosis biomarkers and therapeutic targets for placental insufficiency IUGR.

## Supplementary Information

**Additional file 1.**

**Additional file 2.**

**Additional file 3.**

**Additional file 4.**

**Additional file 5.**

**Additional file 6.**

## Data Availability

Publicly available datasets were analyzed in this study. These data can be found at https://www.ncbi.nlm.nih.gov/geo/query/acc.cgi?acc=GSE114691, https://www.ncbi.nlm.nih.gov/geo/uery/acc.cgi?acc=GSE12216, https://www.ncbi.nlm.nih.gov/geo/query/acc.cgi?acc=GSE24129.

## Abbreviations

IUGR: Intrauterine Growth Restriction
WGCNA: Weighted Gene Co-expression Network Analysis
GEO: Gene Expression Omnibus
DEGs: Differentially Expressed Genes
PPI: Protein-Protein Interaction
STRING: Search Tool for the Retrieval of Interacting Genes
MCODE: The Molecular Complex Detection
DAVID: Database for Annotation, Visualization, and Integrated Discovery
KEGG: The Kyoto Encyclopedia of Genes and Genomes
GO: Gene Ontology
BP: Biological Process
CC: Cellular Component
MF: Molecular Function
