# Supplementary figures and images for: Identification of key genes in pathogenesis of placental insufficiency intrauterine growth restriction
Source: BMC Pregnancy Childbirth. 2022 Jan 28;22:77. doi: 10.1186/s12884-022-04399-3 (PMC8796578; doi:10.1186/s12884-022-04399-3)

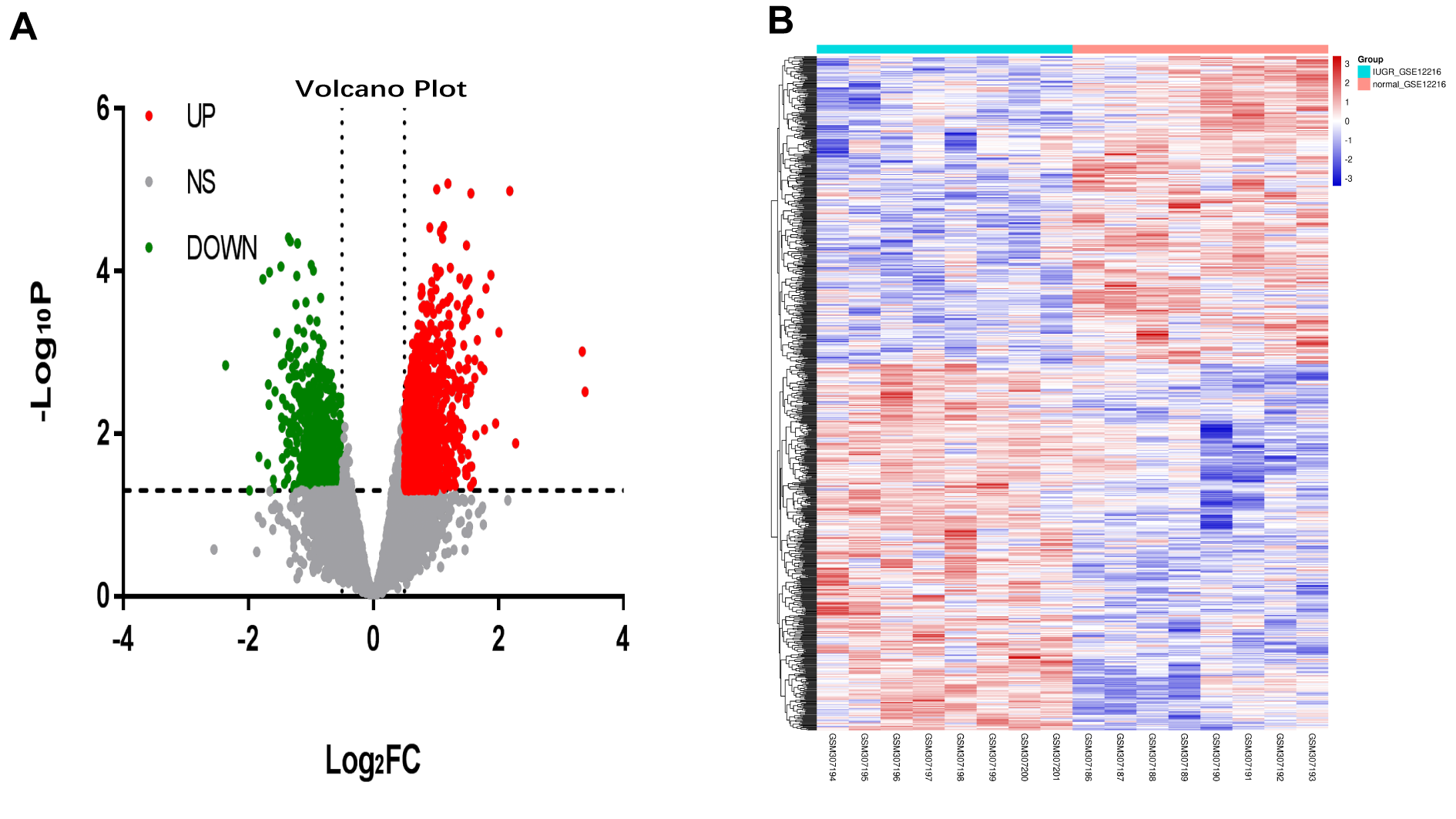

Supplement: Supplementary file 4 — Additional file 4. [file 12884_2022_4399_MOESM4_ESM.tif]

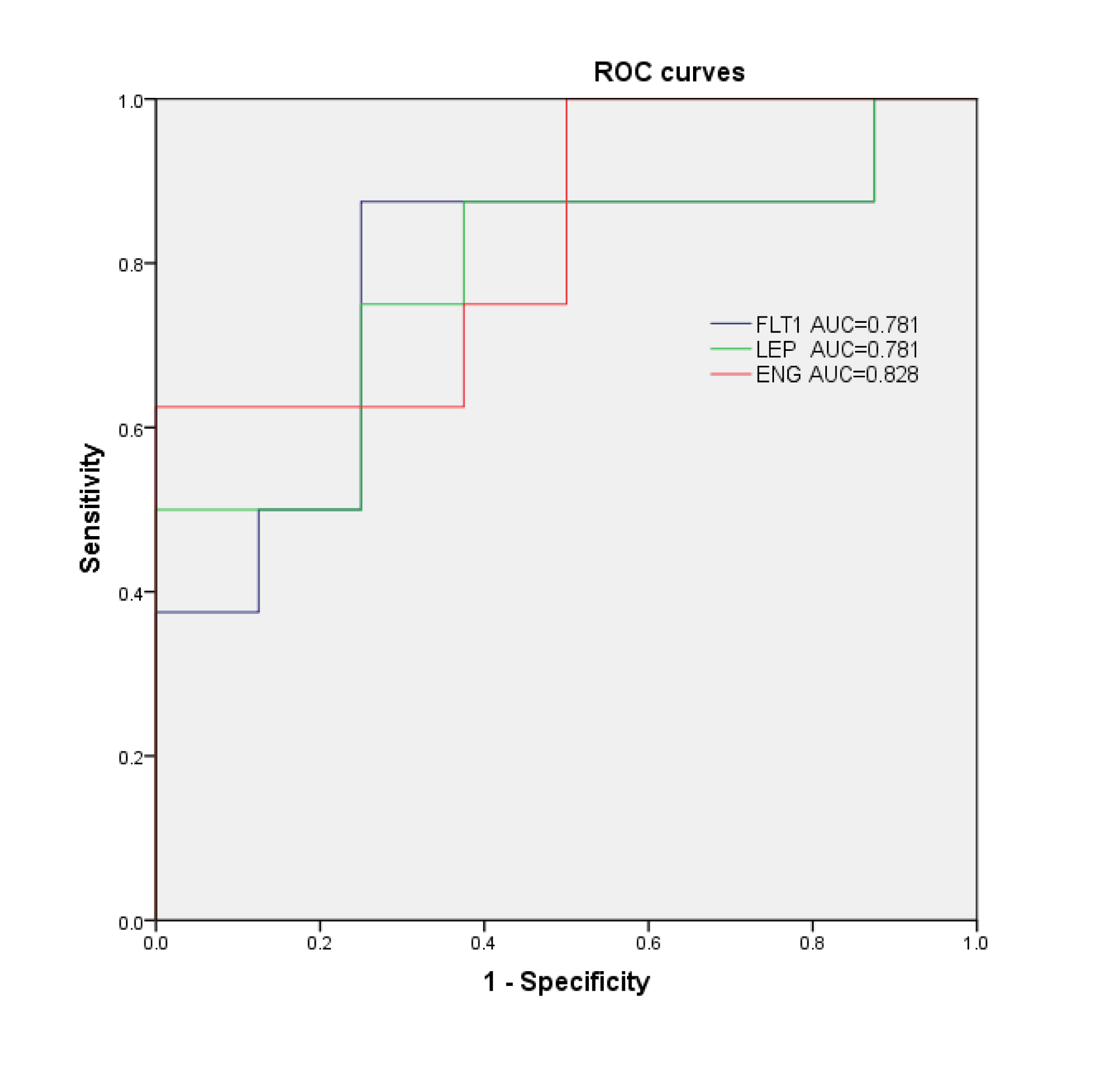

Supplement: Supplementary file 5 — Additional file 5. [file 12884_2022_4399_MOESM5_ESM.tif]

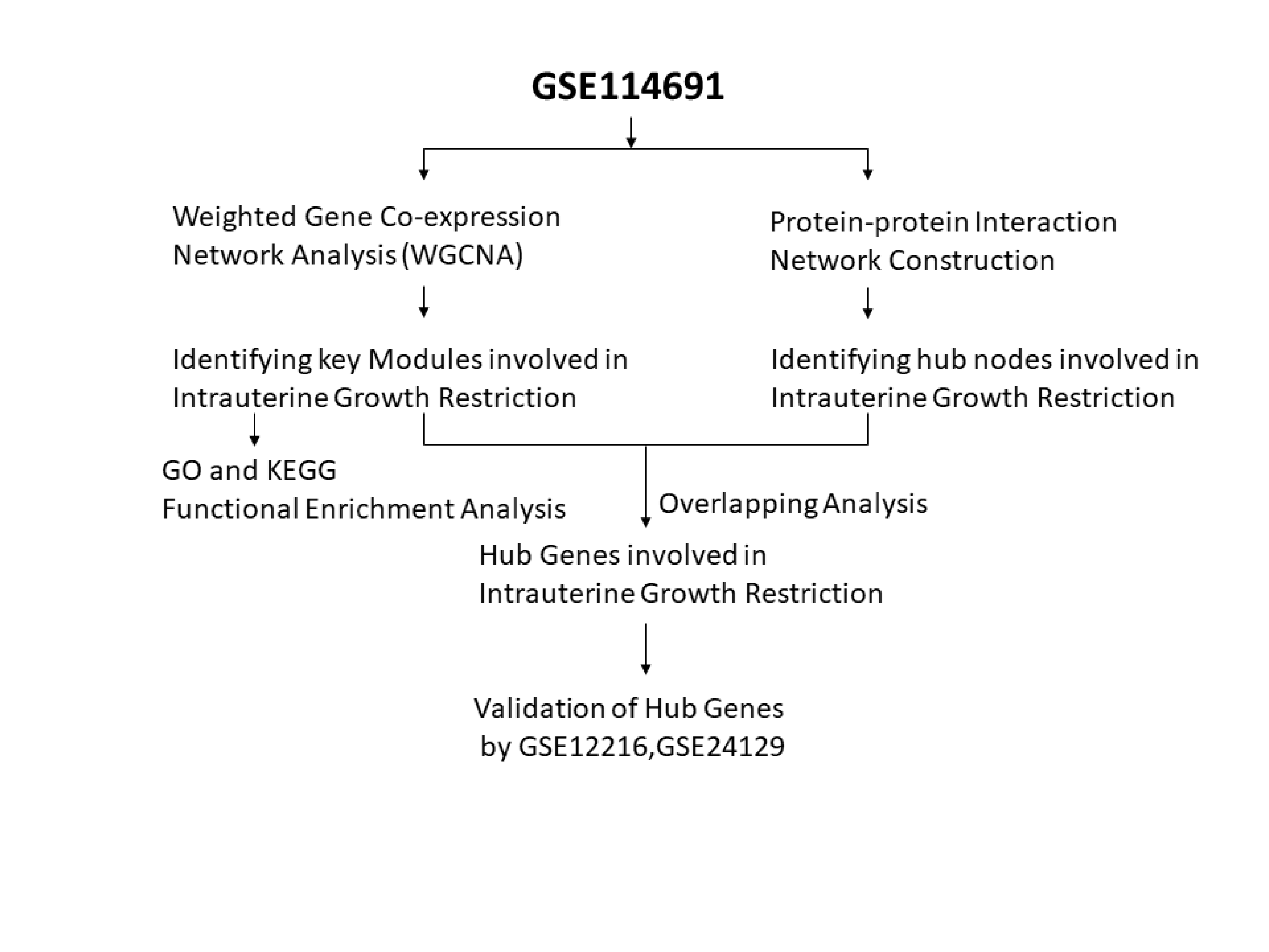

Supplement: Supplementary file 6 — Additional file 6. [file 12884_2022_4399_MOESM6_ESM.tif]
